# Trachoma risk factors in Oromia Region, Ethiopia

**DOI:** 10.1371/journal.pntd.0011679

**Published:** 2023-11-07

**Authors:** Oumer Shafi Abdurahman, Anna Last, David Macleod, Esmael Habtamu, Bart Versteeg, Gebeyehu Dumessa, Meseret Guye, Rufia Nure, Dereje Adugna, Hirpha Miecha, Katie Greenland, Matthew J. Burton

**Affiliations:** 1 International Centre for Eye Health, Clinical Research Department, Faculty of Infectious and Tropical diseases, London School of Hygiene & Tropical Medicine, London, United Kingdom; 2 The Fred Hollows Foundation, Addis Ababa, Ethiopia; 3 Oromia Regional Health Bureau, Addis Ababa, Ethiopia; 4 Environmental Health Group, Department for Disease Control, Faculty of Infectious and Tropical. Diseases, London School of Hygiene & Tropical Medicine, London, United Kingdom; 5 National Institute for Health Research Biomedical Research Centre for Ophthalmology at Moorfields Eye Hospital NHS Foundation Trust and UCL Institute of Ophthalmology, London, United Kingdom; RTI International, UNITED REPUBLIC OF TANZANIA

## Abstract

**Background:**

Trachoma, the leading infectious cause of blindness, is caused by the bacterium *Chlamydia trachomatis* (Ct). Despite enormous disease control efforts and encouraging progress, trachoma remains a significant public health problem in 44 countries. Ethiopia has the greatest burden of trachoma worldwide, however, robust data exploring transmission risk factors and the association between socio-economic status is lacking from some regions. This is the first study to investigate these factors in this South-Eastern region of Oromia, Ethiopia.

**Methodology/Principal findings:**

A total of 1211 individuals were enrolled from 247 households in Shashemene Rural district in Oromia Region between 11^th^ April and 25^th^ June 2018, of whom 628 (51.9%) were female and 526 (43.4%) were children aged 1–9 years. Three standardised ophthalmic nurses examined each participant for the presence of active trachoma using the WHO simplified trachoma grading system. Conjunctival swab samples were collected from the upper tarsal conjunctiva of the left eye of each participant. Ct was detected using quantitative PCR. Risk factor data were collected through structured interviews and direct observations. Clinical signs of trachomatous inflammation-follicular among children aged 1–9 (TF_1-9_) were observed in at least one eye of 106/526 (20.2%) and trachomatous inflammation-intense among children aged 1–9 (TI_1-9_) were observed in at least one eye of 10/526 (1.9%). We detected Ct by PCR in 23 individuals, of whom 18 (78.3%) were in children aged 1–9 years. Among the 106 children aged 1–9 years with TF, 12 (11.3%) were Ct PCR positive and among 20 children aged 1–9 years with TI, 4 (20.0%) were Ct PCR positive. In a multivariable model, adjusting for household clustering, active trachoma was associated with younger age, the poorest households (aOR = 2.56, 95% CI 1.21–5.51), presence of flies on the face (aOR = 2.87, 95% CI 1.69–6.46), and ocular discharge (aOR = 1.89, 95% CI 1.03–3.24). Pre-school children face washing more than once a day had lower odds of having active trachoma (aOR = 0.59, 95% CI 0.19–0.84). The same was true for washing children’s clothing at least once per week (aOR = 0.27, 95% CI 0.33–1.02).

**Conclusion/Significance:**

Younger age, personal hygiene in this age group (presence of ocular and nasal discharges, infrequent washing of faces and clothing) and fly-eye contacts are potential risk factors for trachoma in this setting, suggesting that hygiene interventions and environmental improvements are required to suppress transmission to ensure sustained reduction in disease burden Further studies are needed to evaluate these interventions for trachoma control and elimination. Trachoma remains a disease associated with lower socio-economic status, emphasising the need for continued implementation of control measures in addition to poverty reduction interventions in this region.

## Author summary

Trachoma, caused by ocular Ct, is targeted by the World Health Organization (WHO) for elimination by 2030. The WHO recommends the SAFE strategy to be implemented in all endemic countries. Ethiopia bears the highest trachoma disease burden globally. In Oromia, the biggest region of Ethiopia, the prevalence of trachomatous inflammation-follicular (TF) among children age 1–9 (TF_1-9_) was >30% in 46% of the 2016 GTMP surveyed districts. We conducted a cross-sectional survey of 247 households in Shashemene Rural district in southeastern region of Oromia to explore risk factors for trachoma in this setting. Prevalence of trachomatous inflammation-follicular (TF) was 9.3% overall and 20.2% among children aged 1–9 years. Ct infection among individuals with active trachoma (TF and/or TI) was 12.7% overall. Seventy-eight percent (18/23) of those with Ct detected were children aged 1–9 years. In this setting, active trachoma was associated with younger age, the poorest households, presence of flies on the face, and ocular discharge, emphasizing the importance of the continued implementation of SAFE strategy in addition to poverty reduction interventions.

## Introduction

Trachoma is the leading infectious cause of blindness [1]. As of 2022, 125 million people live in districts where the prevalence of active trachoma in children 1–9 years is at a level requiring population-wide control interventions. More than 67 million of these people live in Ethiopia [2]. Trachoma begins in childhood with repeated infection by the bacterium *Chlamydia trachomatis* (Ct), which triggers chronic conjunctival inflammation, leading to conjunctival scarring. This scarring can cause eyelashes to turn inward and rub on the eye (trichiasis), eventually causing irreversible blinding corneal opacification in adulthood [3]. The WHO recommends that countries implement the SAFE strategy: S—Surgery for trichiasis, A–Antibiotic (mass drug administration [MDA] with oral Azithromycin to whole communities to treat active infection) and F&E—Facial cleanliness and Environmental improvement to suppress transmission [4].

Progress has been made in the prevention and control of this disease. Thirteen countries have been validated by WHO to have achieved elimination in 2022, with three additional countries reporting to have achieved elimination goals [5] Despite these achievements and the continued collective efforts, trachoma remains a public health challenge, particularly in hyperendemic and formerly hyperendemic communities.

Implementation of SAFE, particularly annual MDA with azithromycin, has been successful in reducing infection in many endemic communities in Ethiopia [6]. However, multiple studies have shown that Ct infection and active trachoma can re-emerge in some settings in Ethiopia and in other countries after stopping MDA [7]. Sustained infection control and disease elimination is thought to require suppression of transmission. A better understanding of Ct transmission and the identification of risk factors associated with this and active trachoma in different environments can help inform control approaches. Cross-sectional surveys conducted in trachoma-endemic countries have previously reported active trachoma to be associated with dirty faces, fly-eye contact, limited water access, lack of latrines and crowded living conditions [8–12].

Trachoma risk factor surveys conducted in Ethiopia are mostly concentrated in the Amhara region where there has been a long-standing trachoma control program. Control programs in Oromia, the largest region in Ethiopia, started after the 2016 Global Trachoma Mapping Project (GTMP) data became available. There has been a paucity of epidemiological risk factor data on trachoma from Oromia thusfar. Parts of this region are distinct from Amhara, particularly the Rift Valley area, with almost homogenous demography, religion, and culture. The current study presents an analysis of risk factors for active trachoma from the Rift Valley area of south-eastern Oromia.

## Methods

### Ethics statement

This study was conducted in accordance with the Declaration of Helsinki. It was approved by the National Research Ethics Review Committee at the Federal Ministry of Science and Technology of Ethiopia (Reference, 3-10/001/2018) and London School of Hygiene & Tropical Medicine Ethics Committee (Reference, 14325). Informed written consent was obtained from all individuals above the age of 18. For all individuals below the age of 18 years, written consent was obtained from their parents/guardians and written assent was also obtained from those aged 7 to 17 years.

### Study design and population

This cross-sectional study forms part of the “Stronger SAFE” trachoma research programme, a large collaborative multi-disciplinary set of interrelated studies being conducted in the Oromia Region of Ethiopia to develop and test enhanced A, F & E interventions for trachoma elimination. The aim of this survey was to identify the main risk factors for infection with Ct and active trachoma. It was conducted in Shashemene rural district between 11^th^ April and 25^th^ June 2018. A group of 247 households from a geographically contiguous area were selected within Shashemene rural woreda, chosen because it was an area that had been identified during the 2016 GTMP surveys as having a high prevalence of TF_1-9_ (>40%) [13]. For a household to be included in this survey at least one child aged 1–9 years had to be resident on the day of enumeration. All members of the household were eligible to participate. The detailed survey methodology has been published elsewhere [14].

### Survey

A pre-piloted questionnaire was conducted in each enrolled household. Household location was determined by GPS. Household level risk factor data was collected using a structured questionnaire administered to the primary caregivers to capture socio-demographic and household characteristics, routines and sleeping and hygiene practices hypothesised to be risk factors for trachoma. Information on water supply, availability and use in the dry and rainy seasons was obtained through self-report and direct observation. Data on usual adult and child hygiene and defecation practices were reported by the respondent, whilst latrine availability and use, and the presence of human faeces in the home and compound were directly observed. Survey questions on the water and sanitation situation were based on our previous work [15] and the JMP service ladders for drinking water and sanitation [16]. Household socio-economic status (SES) was measured using eight housing characteristic variables (e.g., number of rooms, construction materials used for wall, roof, and floor), nine utilities and access to infrastructure variables (electricity, kitchen, animal dwelling, water source, latrine ownership and utilisation) and household durable assets (ownership of radio, cell phone, and sleeping bed) and animals (oxen, cows, donkeys, goats, sheep, and chicken). These data were collected by direct observation, with response categories informed by previous studies in this area [17].

Individual-level data were collected from all members of the enrolled households, excluding temporary residents. Temporary residents were defined as individuals who had lived in the household for less than one month. Facial cleanliness was recorded by observing for the presence of ocular and nasal discharge as instructed by our standardised operating procedure (SOP), observed during direct, frontal observation of the child’s face in natural daylight. We defined ocular discharge as the presence of clear or cloudy fluid or dry matter on the eyelid margin or eyelid (including the corners) and nasal discharge as the presence of wet or dry discharge visible outside the nares. The presence of flies on the face was recorded and defined as any fly present on the face during examination by observing the child for 30 seconds in natural daylight, avoiding both full sunshine and full shade. Data collection teams were trained using detailed SOPs.

### Clinical examination and conjunctival sample collection

Three standardised ophthalmic nurses examined the eyes of each household member for active trachoma defined as the presence of trachomatous inflammation–follicular (TF) and / or trachomatous inflammation–intense (TI) in at least one eye, using the WHO simplified trachoma grading system [18,19]. Two conjunctival swab samples were collected from the upper tarsal conjunctiva of the left eye using the following procedure: a sterile swab was wiped four times across the everted tarsal conjunctival surface, rotating the head of the swab by a quarter with each sweep. The swabs were stored in 500 μL of 0.2 M-sucrose-phosphate (2SP) transport medium. One control swab in every 50 conjunctival swabs was randomly selected and collected by holding the swab in the air for 20 seconds, without touching any surface, in the same location that clinical samples were collected. All samples were placed immediately in a cool box with ice packs in the field and then transferred to a -20°C freezer for storage later the same day. There was a maximum of two weeks of storage at -20°C, before onward transfer and storage at -80°C. The DNA extraction and Ct quantitative PCR detection method has been previously published [14]. In brief, samples were classified as Ct PCR positive if amplification of the omcB or pORF2 target was detected within 40 cycles.

### Statistical analysis

Data were collected electronically using Open Data Kit, then cleaned and analysed using STATA 16 (STATA corporation, College Station, Texas USA) [20,21]. We defined active trachoma as presence of TF and/or TI in at least one eye. Age was categorized into 1–5, 6–9, 10–14, and ≥15 years. To determine household SES, asset-based indicator data were analysed using principal component analysis (PCA) using a standard methodology [22–24]. Assets owned by less than 5% or more than 95% of the households were excluded from the analysis. A total of 26 asset variables were included in the final PCA model. The factor score from the first principal component were taken to categorise households into thirds: least poor (wealthiest), middle and poor (poorest). Additional details can be found in S1 Appendix. Water availability in the home is split into more or less than 20 litres. This categorisation and cut-off was informed by the observed frequency and quantity of water present in the household. Variables on usual frequency of (self)-reported adult and child body and clothes washing in the dry and rainy seasons had eight response categories (never, 1–3 times a year, every 2–3 months, once a month, 2–3 times a month, once a week, 1–3 times a week and once a day). These variables were recategorized into binary variables 1–3 times a week vs. less frequent washings based on their frequency

The prevalence of active trachoma was calculated within each category of exposure variable. To estimate the association between each exposure and active trachoma, a mixed effect logistic regression model was used, with active trachoma as the outcome and including a random effect for household to account for within-household clustering. First, each exposure was included in the model separately, only adjusted for age and sex a priori. If the estimate for this association resulted in a p-value less than 0.15 or an odds ratio less than 0.5 or greater than 2 then a second, fully adjusted, analysis was performed. Each exposure that met these criteria were adjusted for all the other exposures that met the criteria unless the exposure [1] introduced collinearity into the model or [2] was believed to lie on the causal pathway between the exposure of interest and active trachoma.

## Results

### Characteristics of study participants and households

**Fig 1:**
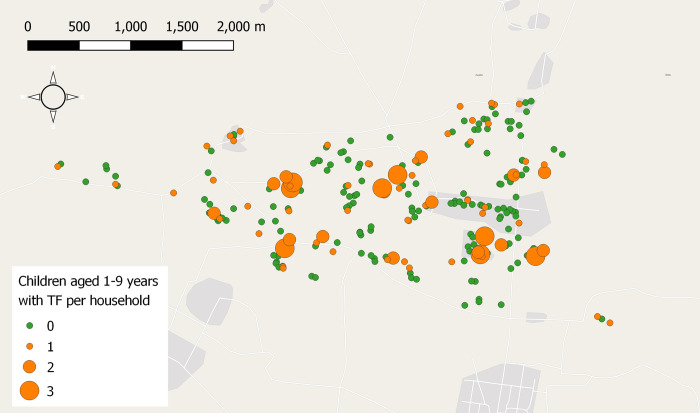
Map of the study area showing the number of children aged 1–9 years with TF in selected households. Note: Each circle represents a household and orange with size [1–3] represent the number of children with disease in each household. Map base layers taken from OpenStreetMap. https://www.openstreetmap.org/copyright generated in QGIS.

Data were collected between April–June 2018. We enrolled 1211 individuals from 247 households, of whom 628 (51.9%) were female and 526 (43.4%) were children aged 1–9 years. Of the primary caregivers who completed the questionnaire, 45.7%(113/247) were female. The geographical distribution of these households is shown in Fig 1. Household level socio-demographic, water access, hygiene and sanitation data are presented in Table 1. Latrine coverage (defined as presence of latrine accessible to the household regardless of the ownership) was 57.5%. 57.8% of adults reported use of a latrine for defecation and 70.0% of the youngest children of the households practice open defecation. The poorest households had less water stored on site compared to the wealthier households (Table 1).

**Table 1:** Household level socio-demographic characteristics, water access, hygiene, and sanitation practices.

Household Characteristic	n/N	(%)
Socio-demographic Characteristics		
Socio-economic status of household (MV = 1)		
Wealthiest	73/246	(29.7)
Middle	82/246	(33.3)
Poorest	91/246	(37.0)
Religion (MV = 1)		
Muslim	242/246	(98.4)
Protestant Christian	3/246	(1.2)
Wakefata	1/246	(0.4)
Compound shared with another household (MV = 1)	90/246	(36.6)
Number of rooms for sleeping space, >2 rooms (MV = 1)	37/246	(15.0)
Mother / Primary care-givers literate (MV = 1)	60/246	(26.8)
Water Availability		
Enough water to meet needs (MV = 1)		
Rainy season	231/246	(93.9)
Dry Season	49/246	(19.9)
Main water source during rainy season (MV = 1)		
Rain	67/246	(27.2)
Piped water in yard/plot	8/246	(3.3)
Public tap/standpipe	120/246	(48.8)
Surface (river)	33/246	(13.4)
Dug well (unprotected)	14/246	(5.7)
Main water source during dry season (MV = 1)		
Public tap/standpipe	176/246	(71.5)
Surface (river)	49/246	(19.9)
Piped water in yard/plot	2/246	(0.8)
Spring well (unprotected)	11/246	(4.5)
Water purchase during dry season (MV = 2)	185/245	(75.5)
Household with any amount of Water available (observed)	225/247	(91.0)
Time to collect water during dry season >30 minutes (MV = 1)	118/245	(48.2)
Amount of water stored in household (observed) >20 litres (MV = 1)		
Wealthiest	48/73	(65.8)
Middle	49/82	(59.8)
Poorest	31/91	(34.1)
Face and Body Washing Practices		
Frequency of youngest child face washing by their mother/carer with water only (MV = 11)		
None	8/230	(3.4)
1–5 times/day	228/236	(96.6)
Frequency of youngest child face washing by their mother/carer with water & soap (MV 10)		
None	212/237	(89.5)
1–3 times/day	25/237	(10.5)
Soap present in the household (observed) (MV = 1)	93/246	(37.7)
Adult bathing during rainy season (reported) <1 time / week	93/247	(37.7)
Adult bathing during dry season (reported) <1 time / week	76/247	(30.8)
Clothes and Other Washing Practices		
Washing children’s clothing in rainy season <1 time / week	107/247	(43.3)
Washing children’s clothing in dry season <1 time /week (MV = 1)	197/247	(80.1)
Wash bedding (dry season)		
Never	16/215	(7.4)
1–3 times / year	199/215	(92.6)
Unwashed dishes in the house (observed) (MV = 1)	145/246	(58.9)
Flies on the unwashed dish (observed) (MV = 2)	80/145	(55.2)
Liquid waste disposal		
Inside the compound	180/246	(73.2)
Outside the compound	66/246	(26.8)
Sanitation Access and Practices		
Access to latrine (MV = 1)	144/246	(57.5)
Access to shared latrine (from those who have access)	26/144	(18.1)
Observed latrine use (from those who have access) (assessed by sight, smell, other signs of use)	113/144	(78.5)
Observed latrine use (from total population) (MV = 1)	113/246	(45.9)
Human faeces observed in the compound (MV = 1)	72/246	(29.3)
Human faeces observed in the house (MV = 2)	7/245	(2.9)
Youngest child usual defecation location (reported) (MV = 1)		
Latrine	73/246	(29.7)
Open defecation–outside the compound	72/246	(29.3)
Open defecation–inside the compound	101/246	(41.1)
Usual adult defecation location (reported) (MV = 1)		
Latrine	142/246	(57.7)
Open defecation–outside the compound	96/246	(39.0)
Open defecation–inside the compound	8/246	(3.3)

All data are self-reported by the respondent unless otherwise specified. MV = Missing Value

### Active trachoma and Chlamydia trachomatis infection

**Fig 2:**
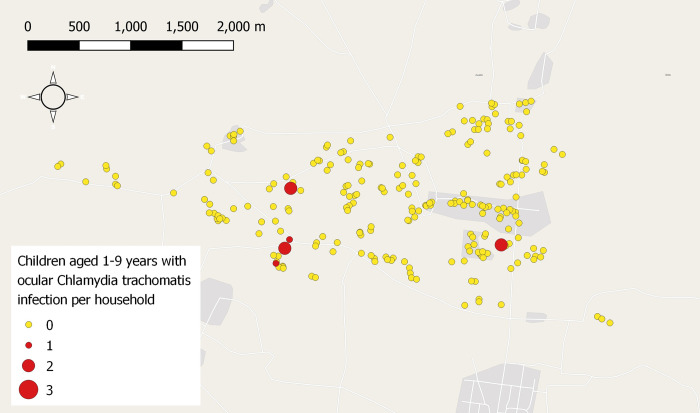
Map of the study area showing the number of C. trachomatis Ct PCR positive samples in selected households. Note: Each circle represents a household and red with size [1–3] represent the number of children with Ct infection in each household. Map base layers taken from OpenStreetMap. https://www.openstreetmap.org/copyright generated in QGIS.

TF was observed in 111/1206 (9.2%) individuals of all ages, and 106/526 (20.2%) children aged 1–9 years. Prevalence of TF was highest in children aged 1–5 years (78/272 [28.8%]) (Table 2). The individuals with active trachoma were found in 85 households, Fig 1. *C*. *trachomatis* was detected 23/1211 (1.9%) ocular swab samples from individuals who lived in 13 households (Fig 2); the large majority of these were from children aged 1–9 years (18/526 [3.4%]), Table 2. Among the 110 children 1–9 years with active trachoma, 15 (12.7%) were PCR positive. At the time of examination ocular discharge, nasal discharge and flies were present on the faces of 336/1207 (27.8%), 278 (23.0%) and 444 (36.8%) participants, respectively.

**Table 2:** *Chlamydia trachomatis* PCR positive samples and active trachoma, by age group.

Age groups	TF or TI in at least one eye (MV = 5)		TF in at least one eye (MV = 5)		TI in at least one eye (MV = 6)		Ct PCR positive (MV = 4)	
	n/N	(%)	n/N	(%)	n/N	(%)	n/N	(%)
1–5 years	81/272	(29.8)	78/272	(28.8)	13/271	(4.8)	9/272	(3.3)
6–9 years	29/254	(11.4)	28/254	(11.0)	5/253	(1.9)	9/254	(3.5)
10–14 years	4/165	(0.2)	3/165	(1.8)	1/165	(0.6)	3/165	(1.8)
≥15 years	4/516	(0.01)	2/516	(0.0)	2/516	(0.4)	2/516	(0.4)
All ages	118/1207	(9.8)	112/1207	(9.3)	21/1205	(1.7)	23/1207	(1.9)
1–9 years	110/526	(20.9)	106/526	(20.2)	18/526	(3.4)	18/526	(3.4)

*MV = Missing values

### Risk factors for active trachoma

In the univariable analysis there was a strong association between active trachoma and the younger age category, the detection of Ct by PCR adjusted for age(aOR = 33.53, 95% CI 8.67–129.60), and–adjusted for sex and age, the presence of ocular discharge (aOR = 3.26, 95% CI 1.89–5.62) nasal discharge(aOR = 2.45, 95% CI 1.40–4.30), and flies on faces (aOR = 4.53, 95% CI 2.45–8.39) (Table 3). There appeared to be an association between SES and active trachoma with lower odds of active trachoma in the “wealthiest” group and higher odds in the middle and poorest groups (p = 0.039. aOR = 2.60, 95% CI 1.22–5.56) (Table 4). There were also associations between active trachoma and less frequent face washing and clothes washing, but not household water availability (Table 5). Adult open defecation was also slightly associated with increased active trachoma (p = 0.0678, OR = 3.24, 95% CI 0.87–12.00).

**Table 3:** Individual level factors associated with the presence of active trachoma, analysed by univariable random effects logistic regression.

Variable	Active trachoma present		Univariable		
	n/N	(%)	OR	(95% CI)	P-value
**Socio-demographic Characteristic**					
Age in years^+^					
1–5	81/272	(29.9)	1		<0.001
6–9	29/254	(11.4)	0.25	(0.14–0.43)	
10–14	4/161	(2.5)	0.04	(0.01–0.13)	
>15	4/512	(0.8)	0.01	(0.00–0.04)	
Gender*					
Female	50/626	(7.9)	1.00		
Male	68/580	(11.7)	1.31	(0.79–2.15)	0.285
Ocular Ct PCR status^**§**^					
Negative	103/1183	(8.7)	1.00		
Positive	15/23	(65.2)	33.53	(8.67–129.59)	<0.001
**Clean Face** ^**§**^					
Ocular discharge					
No	47/870	(5.4)	1.00		
Yes	71/336	(21.1)	3.26	(1.89–5.62)	<0.001
Nasal Discharge					
No	41/928	(4.4)	1.00		
Yes	77/278	(27.7)	2.45	(1.40–4.30)	<0.002
Presence of Flies on the face during examination					
No	21/762	(2.8)	1.00		
Yes	97/444	(21.9)	4.53	(2.45–8.39)	<0.001

^+^ Adjusted for gender

* Adjusted for age

^§^ Adjusted for age and gender

**Table 4:** Household level socio-economic status, housing and water access factors associated with the presence of active trachoma, analysed by univariable random effects logistic regression, adjusted for age and gender.

Variables	Active trachoma		Univariable		
	n/N	(%)	OR	(95% CI)	P-value
**Socio-economic Status**					
least poor (Wealthier)	22/399	(5.5)	1.00		
Middle	44/404	(10.9)	2.24	(1.03–4.84)	
Poorest	52/403	(12.9)	2.60	(1.22–5.56)	0.039
Mother / Primary care-givers literate					
No	89/901	(9.9)	1.00		
Yes	29/305	(6.6)	0.79	(0.41–1.54)	0.498
**Housing Characteristics**					
Living in a shared compound					
No	84/763	(11.0)	1.00		
Yes	34/443	(7.7)	0.55	(0.29–1.01)	0.056
Number of rooms for sleeping space					
≤2 rooms	115/1095	(10.5)	1.00		
>2 rooms	3/108	(2.8)	0.27	(0.06–1.23)	0.090
**Water access and use**					
Amount of water stored in household (observed)					
≤20 litres	67/582	(11.5)	1.00		
> 20 litres	51/624	(8.2)	0.70	(0.39–1.25)	0.230
Time to collect water during dry season					
≤ 30 minutes	62/617	(10.1)	1.00		
> 30 minutes	56/586	(9.6)	0.98	(0.55–1.74)	0.940
Enough water dry season					
No	93/991	(9.38)	1.00		
Yes	25/215	(11.63)	1.48	(0.72–3.05)	0.281
Enough water rainy season					
No	7/55	(12.7)	1.00		
Yes	111/1151	(9.6)	0.69	(0.20–2.35)	0.580

All data are self-reported by the respondent unless otherwise specified.

**Table 5:** Household level hygiene and sanitation factors associated with the presence of active trachoma, analysed by univariable random effects logistic regression, adjusted for age and gender.

Variables	Active trachoma		Univariable		
	n/N	(%)	OR	(95% CI)	P-value
Hygiene Practice					
Frequency of child face washing by their mother/carer with water only					
None	4/37	(10.8)	1.00		
1-5/day	109/1127	(9.7)	0.53	(0.11–2.67)	0.450
Frequency of child face washing by their mother/carer with water and soap					
None	106/1050	(10.1)	1.00		
1–3 times/day	10/121	(8.3)	0.93	(0.35–2.49)	0.890
Presence of soap in the household (observed)					
No Soap	78/765	(10.2)	1.00		
Soap present	40/441	(9.1)	0.96	(0.71–1.31)	0.810
Washing children’s clothing (rainy season)					
1–3 times/week	61/672	(9.1)	1.00		
<1–3 times/week	57/534	(10.7)	1.37	(0.78–2.43)	0.270
Adult bathing during rainy season (reported)					
<1bathing/week	44/456	(9.7)	1.00		
At least once a week	74/750	(9.9)	1.01	(0.56–1.82)	0.980
Adult bathing during dry season (reported)					
<1/week	33/374	(8.8)	1.00		
Once a week	85/832	(10.2)	1.32	(0.79–2.45)	0.380
Frequency children’s clothes washing during the dry season (reported)					
1 times/week	97/892	(10.9)	1.00		
<1 times/week	20/307	(6.5)	0.39	(0.19–0.81)	0.012
Frequency of children’s clothes washing during the rainy season (reported)					
1 times/week	61/672	(9.1)	1.00		
<1 times/week	57/534	(10.7)	1.37	(0.77–2.45)	0.280
Wash bedding (dry season)					
Never	10//82	(12.2)	1.00		
1–3 times / year	103/969	(10.6)	1.02	(0.35–3.00)	0.970
Liquid waste disposal					
Inside the compound	9/126	(7.1)	1.00		
Outside the compound	106/1076	(9.9)	1.64	(0.88–3.05)	0.120
Human faeces observed in the compound					
No	77/831	(9.3)	1.00		
Yes	72/246	(29.3)	1.03	(0.56–1.92)	0.900
Human faeces observed in the house					
No	113/1171	(9.7)	1.00		
Yes	5/32	(15.6)	2.03	(0.39–0.41)	0.860
Latrine access					
No	57/463	(12.3)	1.00		
Yes	61/743	(8.2)	0.60	(0.34–1.08)	0.089
Latrine use (observed)					
No	15/176	(8.5)			
Yes	46/567	(8.1)	1.07	(0.42–2.74)	0.883
Latrine drop hole cover (observed)					
No	56/656	(88.7)	1.00		
Yes	4//76	(11.3)	0.63	(0.15–2.66)	0.530
Youngest child usual defecation location (reported)					
Latrine	24/364	(6.6)	1.00		
Open defecation–outside the compound	53/315	(15.1)	2.35	(1.19–4.65)	0.370
Open defecation–inside the compound	41/491	(8.4)	0.97	(0.50–1.92)	
Adult defecation location (reported)					
Latrine	61/734	(8.3)	1		
Open defecation–outside the compound	50/434	(11.5)	1.45	(0.84–2.51)	0.068
Open defecation–inside the compound	7/38	(18.4)	3.24	(0.87–12.0)	

NB: All data are self-reported by the respondent unless otherwise specified.

In the final multivariable analysis (Table 6), we found that active trachoma was higher in those of younger age category, from poorer households (aOR = 2.58, 95% CI 1.21–5.51), had ocular discharge (aOR = 1.91, 95% CI 1.03–3.24), had flies on the face during examination (aOR = 3.90, 95% CI 1.69–6.46) and less than once per week washing of children’s clothes in the household during the dry season(aOR = 0.27, 95% CI 0.33–1.02). Pre-school children face washing more than once a day had lower odds of having active trachoma (aOR = 0.59, 95% CI 0.19–0.84).

**Table 6:** Multivariable random effects logistic regression model for factors associated with active trachoma, adjusted for household level clustering.

Variable	Active trachoma		Multivariable*		
	n/N	(%)	OR	(95% CI)	P-value
Age in years					
1–5	81/272	(29.9)	1.00		
6–9	29/254	(11.4)	0.34	(0.19–0.60)	<0.001
10–14	4/161	(2.5)	0.08	(0.02–0.23)	
>15	4/512	(0.8)	0.03	(0.01–0.08)	
Gender					
Female	50/626	(7.9)	1.00		
Male	68/580	(11.7)	1.27	(0.77–2.09)	0.344
Socio Economic Status					
Least poor (Wealthiest)	22/399	(5.5)	1.00		
Middle	44/404	(10.9)	2.34	(1.13–5.39)	0.0427
Poorest	52/403	(12.9)	2.56	(1.21–5.51)	
Ocular discharge					
No	47/870	(5.4)	1.00		0.027
Yes	71/336	(21.1)	1.89	(1.03–3.24)	
Flies on the face					
No	21/762	(2.8)	1.00		0.002
Yes	97/444	(21.9)	2.87	(1.69–6.46)	
Frequency children’s clothes washing during the dry season (reported)					
<1 times/week	20/307	(6.5)			
1 times/week	97/892	(10.9)	0.27	(0.19–0.84)	0.005
Frequency of child face washing by their mother/carer with water only (reported)					
None	72/556	(12.9)	1.00		0.077
1-5/day	46/650	(7.1)	0.59	(0.33–1.02)	

*Not adjusted for nasal discharge, latrine use, number of rooms for sleeping, sharing compound, youngest child defecation location, and liquid waste disposal. Additionally, because of the small number of Ct PCR positive samples, we did not adjust for Ct, but did adjust for water and latrine availability.

## Discussion

In this rural community in southeastern Oromia the prevalence of trachoma remains high, with a TF_1-9_ of 20.2%. We observed a strong independent association between being Ct PCR positive and having active trachoma, consistent with other studies [25]. The prevalence of trachoma in our study community was lower than the 2016 GTMP survey demonstrated [13]. This is the first study to report Ct infection data from this area.

There may be several reasons for the difference in prevalence estimates of TF_1-9_ reported in the two studies. Trachoma exhibits clustering at both community and household levels [26–28]. Hence, the relatively small scale and discrete geographical area of our study may underestimate the true prevalence in the wider district. Additionally, the effect of one round of MDA with 94% (241,416/256,906 population) coverage may have had a significant impact, particularly in a discrete geographical area, relatively close to the urban area of Shashemene.

High coverage MDA has been shown to dramatically reduce the prevalence of TF_1-9_ and Ct infection after a single round in other populations in sub-Saharan Africa [29,30]. This is likely to reflect the geographic and social characteristics of these regions, including lower population movement resulting in a reduced likelihood of imported infection. There may also be secular trend, as demonstrated in the study conducted in Nepal in which only 36% of the 53% prevalence reduction was attributed to treatment with antibiotic [31]. Data from The Gambia and Senegal showed a smaller reduction in TF_1-9_ after one round of MDA, from 23.9% to 17.7% and from 14.9% to 8.0%, respectively [32,33]. This may be due to epidemiological differences in these regions, including baseline prevalence of TF_1-9_ prior to MDA programmes commencing, and may also reflect the reduced specificity of TF as an indicator for infection as TF prevalence decreases below 10% [34]. In the Amhara region of Ethiopia, despite multiple rounds of high coverage MDA (>90% in some areas), disease and infection remain high, likely due to the high baseline prevalence of TF_1-9_ in this region [35,36].

The lower prevalence of TF_1-9_ demonstrated in this study supports the hypothesis that Ct transmission returns slowly in the absence of repeated annual MDA. This phenomenon has also been reported in the Gurage region of Ethiopia [37]. In addition to MDA coverage, baseline prevalence, individual treatment efficacy, concurrent implementation of F and E measures, population migration and changes in social demography leading to secular trend may influence continued transmission in the region [38,39]. This is consistent with epidemiological models which suggest the possibility of slow return of reinfection after elimination is achieved [40]. If the return of infection is slow, then there may be opportunities to interrupt transmission using sustained F and E measures.

Most households in this community (91%) were observed to have some water stored in their household, and 52% had >20 litres of water. Water availability in this community appears to be better than in some neighbouring districts, where water scarcity is common at times of the year, so may not be a major barrier to face washing interventions. In 21 households (8.5%) where there was scarcity of water (observed to have no water during the visit), there was no association between water availability and active trachoma, but number of households where this was true was small and therefore may not be representative of regions with genuine water scarcity. This was a cross sectional survey and water scarcity may also be associated with seasonality. Limited water access has been shown to be associated with active trachoma in other studies [41]. Additionally, limited water supply, and perceived water scarcity, is a common reason for not washing faces regularly and greater availability of water in the household is associated with a lower likelihood of having active trachoma [42,43].

Less frequent washing of young children’s clothes during the dry season (<1 times/week) was associated with active trachoma. This association supports the findings from our previous study, where we detected Ct on extraocular surfaces including the cuffs and neckline of children’s clothing, suggesting the need to place emphasis on hygiene practices to interrupt trachoma transmission in the context of the SAFE strategy [14].

Several other hygiene and environmental sanitation factors were associated with active trachoma in this environment. These findings are consistent with other published trachoma risk factor studies conducted elsewhere [25,44–47]. Ocular and nasal discharge and the presence of flies on the face were strongly associated with active trachoma [25,33,41,47–49]. It is likely that the ocular and nasal discharge present in eyes with active trachoma attract eye-seeking flies to feed on secretions, allowing them to pick up Ct and potentially act as a passive vector for trachoma. We know from our field observations, that flies also land on clean faces, particularly at times of high fly population density, supporting a potential role for these flies in the transmission of trachoma in this environment.

Poor environmental sanitation could favour larger fly populations and more frequent fly-eye contacts, promoting transmission–a phenomenon previously described in The Gambia [50]. This may suggest that simple improvements in environmental sanitation practices might reduce fly breeding sites and help suppress transmission. A longitudinal study in the Gambia found that simple pit latrines can reduce fly populations [51]. The Flies and Eyes trial, a three-arm study conducted in The Gambia, recorded fewer fly-eye contacts in the latrine arm villages compared to the control arm, with no extra latrine provision [52].

Neither latrine coverage nor utilization was associated with a reduction in active trachoma. This supports study findings elsewhere suggesting that a threshold effect exists to reduce transmission and thus prevalence of active trachoma in communities [49].

We found an association between living in the poorest households and having active trachoma. This is the first study in the Oromia region to systematically investigate the association between active trachoma and SES. These findings are consistent with an earlier study from the Amhara Region of Ethiopia, which found a strong relationship between late-stage trachoma (trichiasis) and lower asset-based assessment of SES [53]. This association could have several explanations. Compared to the wealthiest households, the poorest households in this study had less water observed in the household at time of our visit and therefore possibly less available, or allocated, for face washing. The wealthiest households were more likely to have latrines and soap in their household than the poorest households. Interestingly, even after adjusting for latrine availability and hygiene practices (particularly presence of ocular discharge and having at least 5L litres of water at home) we found an association between active trachoma and SES, confirming that trachoma is a disease that disproportionately affects the poorest communities.

Our study has several limitations. It is cross sectional, so only provides a ‘snapshot’ of data in time. This study design used geographical sampling methods rather than the probability proportional to size method, and therefore provides only an estimate of prevalence of trachoma in this small geographical area, which may be affected by the clustered nature of trachoma. Formal tests of agreement between graders for ocular discharge and nasal discharge were not conducted. However, we made the best effort to ensure the consistency in results through training and pilot study. The sample size is small, which should be taken into account in the interpretation and generalizability of results. Many of the risk factors investigated involved self-report of complex hygiene behaviours and may have been subject to social desirability and recall bias. However, the study benefitted from direct observation of proxies for behaviour and other environmental variables, which strengthens the methodology. Additionally, these findings are supported by similar findings elsewhere in Ethiopia [49,54–57].

## Conclusion

The risk factors for trachoma in this region are consistent with the findings from other studies in Ethiopia and sub-Saharan Africa. Specifically, active trachoma was associated with younger age, the presence of ocular and nasal discharge, poor environmental sanitation (favouring fly breeding), fly-eye contacts during times of high fly population density and poorer households. To control trachoma in this region, the transmission dynamics involving flies, Ct infection and hygiene need to be better understood. Further studies are needed to understand the role of flies and face washing (personal hygiene) in transmission. Interventions to suppress transmission by these routes are currently being evaluated in a cluster randomized controlled trial testing alternative antibiotic schedules and enhanced F and E intervention packages (Stronger SAFE, https://www.iscrtn.com/ISRCTN40760473).

## Supporting information

S1 Appendix Additional details on the principal component analysis to determine household socio economic status. (DOCX)
