# Supplementary material for: Trachoma risk factors in Oromia Region, Ethiopia
Source: PLoS Negl Trop Dis. 2023 Nov 7;17(11):e0011679. doi: 10.1371/journal.pntd.0011679 (PMC10629622; doi:10.1371/journal.pntd.0011679)
Supplement: S1 Appendix — (DOCX) [file pntd.0011679.s001.docx]

Appendix 1. Principal component analyses (PCA)

A total of 26 asset variables were included in the final PCA model. The factor score from the first principal component was taken to categorise households into thirds: least poor (wealthiest), middle and poorest.

Descriptive analysis to decide on variables to be included in the PCA was conducted. Then we analysed each category separately to better understand the data and see whether clumping and truncation would be an issue. We then conducted the final PCA analysis by adding all the variables and saw what the distribution looks like and the Keiser-Meyer Olkin (KMO) analysis to test if the variables are appropriate for factor analysis. Our data had an overall KMO score of 0.67, which is considered sufficient for PCA analysis of socio-economic status. Eigenvalues associated with PC1 was 5.88 and the share of variance associated with PC1 = 0.23%.

Assets owned by less than 5% or more than 95% of the households were excluded from the analysis^(^[^1^](#_ENREF_1)^)^. Variables used in the PCA were having access to electricity, number of rooms for sleeping space, total number of rooms in the household, wall made of wood and mud, wall made from only mud, wall made from only wood, having tin roof, having grass roof, having plastic roof, having kitchen in the main room, having separate kitchen in the main room, having separate kitchen outside of the main living room, having separate animal room, source of water (public piped tap, river), owning latrine, type of latrine (pit latrine vs other poorly constructed latrines), owning radio, cell phone or sleeping bed, owning animals (oxen, cows, donkeys, calves, sheep, goats, chicken).

Our analysis involving SES used the categorization derived from the first principal component as a single variable.

1. Habtamu E, Wondie T, Aweke S, Tadesse Z, Zerihun M, Zewdie Z, et al. Trachoma and Relative Poverty: A Case-Control Study. PLoS neglected tropical diseases. 2015;9(11):e0004228.
